# Protection by Anti-β-Glucan Antibodies Is Associated with Restricted β-1,3 Glucan Binding Specificity and Inhibition of Fungal Growth and Adherence

**DOI:** 10.1371/journal.pone.0005392

**Published:** 2009-04-28

**Authors:** Antonella Torosantucci, Paola Chiani, Carla Bromuro, Flavia De Bernardis, Angelina S. Palma, Yan Liu, Giuseppina Mignogna, Bruno Maras, Marisa Colone, Annarita Stringaro, Silvia Zamboni, Ten Feizi, Antonio Cassone

**Affiliations:** 1 Department of Infectious, Parasitic and Immune-mediated Diseases, Istituto Superiore di Sanità, Rome, Italy; 2 Glycosciences Laboratory, Faculty of Medicine, Imperial College London, London, United Kingdom; 3 Department of Biochemical Sciences ‘A. Rossi Fanelli’, University of Rome ‘La Sapienza’, Rome, Italy; 4 Departments of Technology and Health, Istituto Superiore di Sanità, Rome, Italy; 5 Department of Therapeutic Research and Medicine Evaluation, Istituto Superiore di Sanità, Rome, Italy; Pasteur Institute, France

## Abstract

Anti-β-glucan antibodies elicited by a laminarin-conjugate vaccine confer cross-protection to mice challenged with major fungal pathogens such as *Candida albicans, Aspergillus fumigatus* and *Cryptococcus neoformans*. To gain insights into protective β-glucan epitope(s) and protection mechanisms, we studied two anti-β-glucan monoclonal antibodies (mAb) with identical complementarity-determining regions but different isotypes (mAb 2G8, IgG2b and mAb 1E12, IgM). *C. albicans*, the most relevant fungal pathogen for humans, was used as a model.

Both mAbs bound to fungal cell surface and to the β1,3-β1,6 glucan of the fungal cell wall skeleton, as shown by immunofluorescence, electron-microscopy and ELISA. They were also equally unable to opsonize fungal cells in a J774 macrophage phagocytosis and killing assay. However, only the IgG2b conferred substantial protection against mucosal and systemic candidiasis in passive vaccination experiments in rodents. Competition ELISA and microarray analyses using sequence-defined glucan oligosaccharides showed that the protective IgG2b selectively bound to β1,3-linked (laminarin-like) glucose sequences whereas the non-protective IgM bound to β1,6- and β1,4-linked glucose sequences in addition to β1,3-linked ones. Only the protective IgG2b recognized heterogeneous, polydisperse high molecular weight cell wall and secretory components of the fungus, two of which were identified as the GPI-anchored cell wall proteins Als3 and Hyr1. In addition, only the IgG2b inhibited in vitro two critical virulence attributes of the fungus, hyphal growth and adherence to human epithelial cells.

Our study demonstrates that the isotype of anti-β-glucan antibodies may affect details of the β-glucan epitopes recognized, and this may be associated with a differing ability to inhibit virulence attributes of the fungus and confer protection *in vivo*. Our data also suggest that the anti-virulence properties of the IgG2b mAb may be linked to its capacity to recognize β-glucan epitope(s) on some cell wall components that exert critical functions in fungal cell wall structure and adherence to host cells.

## Introduction

Diseases caused by fungi are increasingly impacting on the health of populations, particularly constituting a large fraction of health care-associated infections. Subject categories at high risk of fungal infection are cancer patients under immunosuppressive chemotherapy, subjects undergoing major surgery and critically-ill patients under supportive ventilation and bearing central venous and urinary catheters [1]–[3]. Both diagnosis and antifungal therapy are of limited effectiveness in these patients, resulting into treatment failures and associated mortality [2], [4], [5]. In addition, the spectrum of fungal pathogens has enlarged to include yeasts and moulds that are refractory to most antifungals, posing remarkable challenges to infection control measures [1], [3], [6].

In this context, it is much hoped that immuno-prophylactic or -therapeutic treatments will be developed to drastically reduce the incidence of fungal infections and resulting mortality. Toward these goals, antibody-eliciting antifungal vaccines or antibody-based treatments have recently gained particular appeal [7]–[12]. There is now a firm experimental evidence that some antibodies can exert a significant antifungal defensive action [7], [8], [12]–[14]. Experience with anti-bacterial or anti-viral vaccines in current use suggests that vaccines that induce protective antibodies may be more easily generated and standardized as compared to those inducing protection by cell-mediated immunity. On the other hand, development of clinically useful antifungal antibodies may profit from the on-going outstanding advances in recombinant DNA technologies and protein engineering.

Remarkable progress has been made recently in the discovery of fungal antigens that confer antibody-mediated protection. There are several examples of experimental subunit vaccines that are able to prevent some of the most widespread fungal infections through the induction of protective antibodies. Some of these appear particularly promising [12], [15]–[21]. The feasibility of conferring passive protection through administration of protective antibodies, with or without antimycotic therapy, is also being investigated actively. A number of monoclonal or recombinant antifungal antibodies are available, which have proven to be protective in preclinical or clinical models of passive vaccination against some fungal infections [19], [20], [22]–[28].

It is of particular interest that several protective antifungal antibodies appear to provide protection by blocking virulence factors [17], [18], [21], [25], [29]–[31] or by directly affecting fungus growth [20], [23], [32]. This mode of action, which is independent of help by the host immunity, would be particularly advantageous in the setting of the immunocompromized hosts, who are at major risk of severe fungal infections.

Presently, researchers in the field are optimistic that antifungal vaccines and antibodies of clinical usefulness will be soon generated [7]–[9], [11], [12]. For success, however, it is necessary to identify precisely antibodies and antigens that can generate a protective immunity and to elucidate the mechanisms of immune protection, also in consideration of the large variability of protective value among antifungal antibodies with similar or even equal antigen specificity [9], [10], [12], [33]–[35]. Among the most recently described subunit vaccines and antifungal antibodies, those targeting major polysaccharides or polysaccharide-associated proteins of the fungal cell wall have been shown to exert protection in various experimental models of fungal infection, both in normal and in immunocompromized animals [15], [17]–[20], [22], [25], [27]–[29], [36], [37].

In particular, a number of mannan or β-glucan protein conjugates vaccines have shown efficacy in experimental models of candidiasis, aspergillosis and cryptococcosis, the three most prevalent fungal infections of humans [15], [18], [20], [29], [36]. One of these candidate vaccines, composed by laminarin (β1,3-glucan) conjugated with the genetically-inactivated diphtheria toxin CRM197 (Lam-CRM vaccine) has been found to induce the production of anti-β-glucan antibodies capable of conferring protection against all three the above infections, showing for the first time that it is possible to immunize with a single antigen against evolutionarily distant, unrelated infectious agents such as *Candida*, *Aspergillus* and *Cryptococcus* spp. [20 and Vecchiarelli et al, personal communication]. The same broad protective specificity was shown by mAb 2G8, a laminarin-recognizing, anti-β-glucan IgG2b monoclonal antibody, which was able to control infections by *C. albicans and C. neoformans*. [20], [22]. As for other promising antifungal vaccines and antibodies, however, details of the antigenic determinants and effector mechanisms of the protective immunity provided by the β-glucan-based vaccine and anti- β-glucan mAbs remain largely elusive.

In this paper, we have tried to gain insights into the mechanisms of protection induced by anti-β-glucan antibodies by comparing the anti-β-glucan mAb 2G8 with a mAb (named 1E12) which has equal sequences of light and heavy chain Complementarity Determining Regions (CDRs) as the IgG, but is of different isotype (IgM). *C. albicans*, the most widespread agent of fungal disease in humans, has been used as a test model in our investigations.

## Results

### Anti-β-glucan mAb 2G8 and 1E12 and their binding to fungal cell wall

In the search for protective anti-β-glucan antibodies analogous to those elicited by the Lam-CRM vaccine, we generated a number of anti-β-glucan murine monoclonal antibodies. The mAbs 2G8 and 1E12, belonging, respectively, to the IgG2b and the IgM class, were selected for further studies. In preliminary experiments, both mAbs were found to specifically react in ELISA with isolated fungal β-glucans from *C. albicans* or *Saccharomyces cerevisiae*, while not recognizing at all α-linked glucans or fungal (*C. albicans* or *S. cerevisiae*) mannans (data not shown). The deduced amino acid sequences of variable regions of the two mAbs (Table 1) showed a complete identity of their three heavy and light chain CDRs.

**Table 1:** Deduced amino acid sequences of the variable regions of the light (VL) and heavy (VH) chains of IgG mAb 2G8 and IgM mAb 1E12 showing their complete identity.

**IgG mAb 2G8**
**VL**
DIVMTQSPLTLSVTIGQPASISCKSSQSLLYSNGNTHLNWLLQRPGQSPKRLIYLVSKLDSG
VPDRFTGSGSGTDFTLKISRVEAEDLGFYYCVQGTHFPYTFGGGTKLEIKRADAAPTVS
**VH**
LQQSGAELMKPGASVKISCKATGYTLSSYWLEWVKQRPGHGLEWIGEILPGSGSTNYNEK
FKGKATFTADTSSNTAYMQLSSLTSEDSAVYYCAREGWYFDVWGAGTTVTVSSAKTTP
PSVYPLA
**IgM mAb 1E12**
**VL**
DIVMTQSPLTLSVTIGQPASISCKSSQSLLYSNGNTHLNWLLQRPGQSPKRLIYLVSKLDSG
VPDRFTGSGSGTDFTLKISRVEAEDLGFYYCVQGTHFPYTFGGGTKLEIKRADAAPTVS
**VH**
LQQSGAELMKPGASVKISCKATGYTLSSYWLEWVKQRPGHGPEWIGEILPGSGSTNYNEK
FKGKATFTADTSSNTAYMQLSSLTSEDSAVYYCAREGWYFDVWGAGTTVTVSSAKTTP
PSVYPLA

The solid, dotted and dashed underlining indicate CDR 1,2 and 3, respectively.

**Figure 1:**
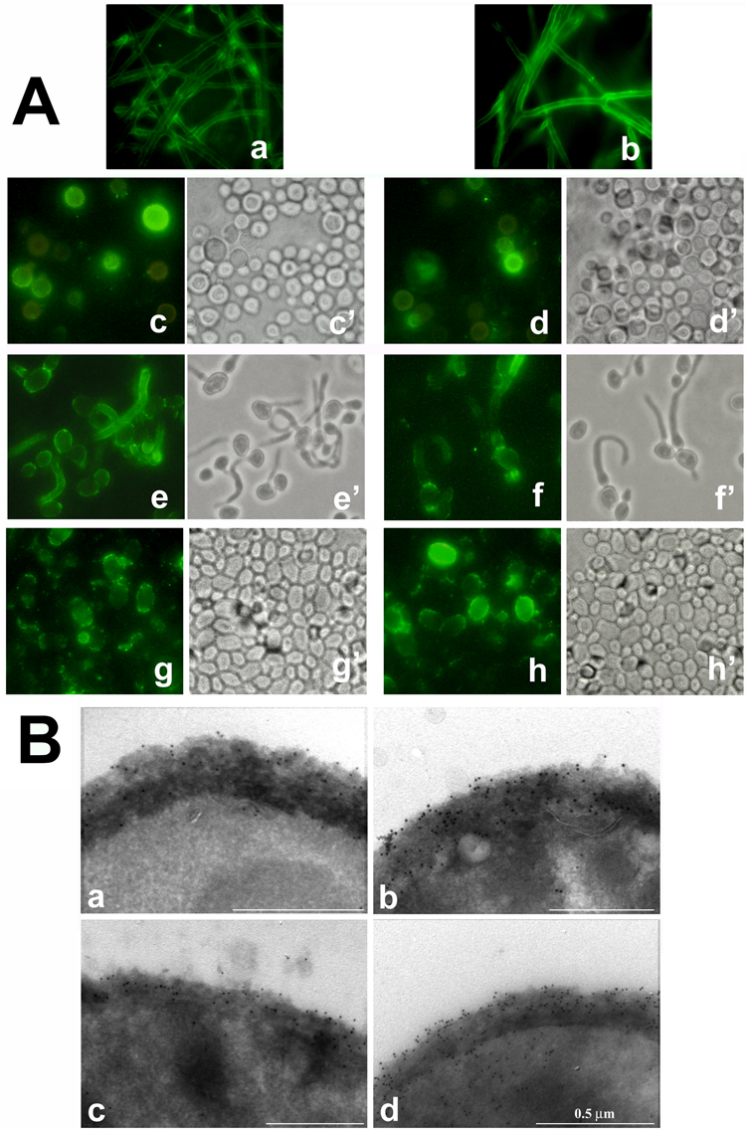
Expression of anti-β-glucan mAb epitopes in major fungal pathogens for humans. Panel A: immunofluorescence staining pattern of hyphal filaments of *Aspergillus fumigatus* (a, b), *Cryptococcus neoformans* cells (c, d) and *C. albicans* germ-tubes (e, f) or yeast cells (g, h) reacted with the IgG (a, c, e, g) or the IgM (b, d, f, h) anti-β-glucan mAb. Sub-panels c′ through h′ show the corresponding bright field images. Magnification: 800× times ( except *A. fumigatus* hyphae, magnified 400× times). Panel B: ultrathin sections from cryofixed yeast (a,b) or hyphal (c,d) cells of *C. albicans* after immunogold labelling with the IgG (a,c) or the IgM (b, d) mAb.

Both antibodies were found to bind to *C. albicans* germ-tubes (hyphal precursors) and *A. fumigatus* hyphae (Figure 1, panel A, a–b; e–f). They also bound to a proportion of poorly encapsulated *C. neoformans* cells (which display β-glucan on their surface) and *C. albicans* yeast cells, although with large cell to cell variations in labelling intensity (Figure 1, Panel A, c–d; g–h).

MAb binding to *C. albicans* cell surface was then examined in more detail by immuno-electron microscopy analysis of criofixed, ultrathin sections, a type of preparation which is believed to optimally preserve cellular components in their native state. Fig. 1, panel B, a–d shows discrete, non uniform levels of gold immunolabelling for both IgG- and IgM-reactive material throughout the thicker cell wall of the yeast and the thinner cell wall of the hyphae. Gold particles were also present at the cell surface of both yeast and hyphal cells, and both in IgM- and in IgG-labelled sections (Figure 1, panel B). Quantitative assessment of the number of gold particles per cell wall area did not reveal statistically significant differences between IgM- and IgG labelling ( data not shown)

### The IgG and the IgM anti-β-glucan mAbs confer different degrees of protection in experimental models of *C. albicans* infection

We have previously reported that the IgG mAb 2G8 is able to control infections by *C. albicans* or *C. neoformans* in different animal models [20], [22]. As in experimental fungal diseases there are a few but well established examples of antibodies whose protective value is modulated depending on the isotype [33], [38], we wondered whether, and to what extent, the anti-β-glucan IgM was also protective. To assess this issue, we carried out comparative protection assays with the two mAbs in different experimental models of *C. albicans* infection.

**Figure 2:**
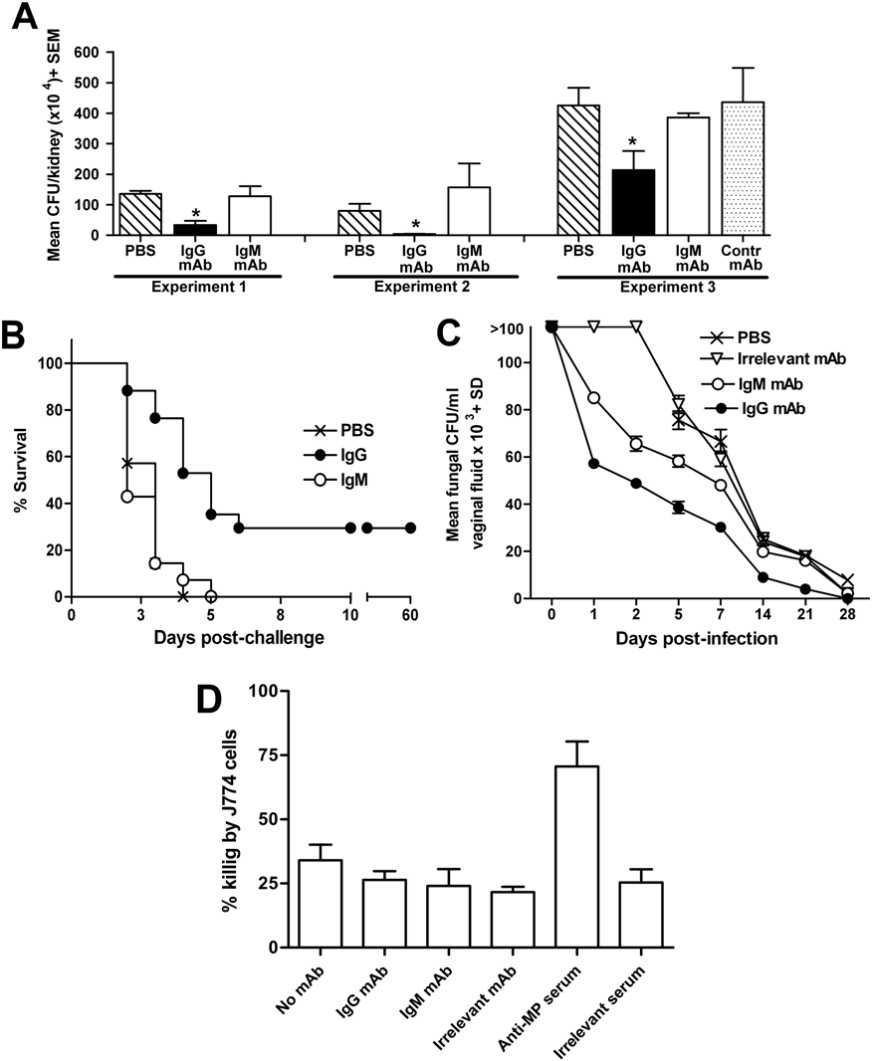
Protection by anti-β-glucan mAbs. Panel A : Fungal burden in kidney following a systemic infection with *C. albicans* in anti-β-glucan mAb-treated mice. In each of these experiments, groups of three mice were passively immunized by the i.p. route with 100 µg/0.5 ml of the IgG or IgM anti-β-glucan mAb, as indicated whereas control mice (three per group) received 0.5 ml of PBS only (Experiments 1 and 2) or 100 µg/0.5 ml of an irrelevant IgG2b mAb (Experiment 3). Two hours post passive immunization, the animals were infected i.v. with *C. albicans* (5×10^5^, Exp 1 and 2 or 10^6^ cells/mouse, Exp 3) and extent of fungal invasion was evaluated at day 2 post-challenge thruogh CFU enumeration in left kidney. The asterisks indicate a statistically significant difference (P<0.05) in mean CFU number/kidney in the corresponding group of animals as compared to the PBS-treated (experiments 1 and 2) or to the irrelevant mAb-treated group (experiment 3). Panel B: Survival of mice given a single, prophylactic administration of the anti-β-glucan mAbs and lethally infected with *C. albicans*. Mice (7 per group) were administered a single dose of the indicated mAb (150 µg/0.5 ml, i.p.) or 0.5 ml of PBS and, 2 h later, received a lethal challenge with *C. albicans* (10^6^ cells/mouse, i.v.). Log rank test indicated statistically significant differences between survival curves of PBS- and IgG- and between those of IgG- and IgM-treated animals, but no significant difference between PBS- and IgM- treated mice. Panel C: Protection by the anti-β-glucan mAbs in a rat model of vulvovaginal candidiasis. The graph shows kinetics of fungal clearance from the vagina (mean+SE values of *C. albicans* CFU in vaginal fluids at the indicated times post-infection) in oophorectomized, estrogen-treated rats (five per group) intravaginally infected with *C. albicans* and treated with the anti-β-glucan mAbs or with an irrelevant mAb (40 µg/200 µl at 1, 24 and 48 hours post-infection) or with PBS alone (200 µl, same schedule). The experiment was repeated twice with similar results. Panel D: Evaluation of the opsonic activity of the mAbs. *C. albicans* killing by J774 murine macrophages was assessed by a classical CFU count after 3 h of contact (MOI 0.2∶1) in the absence or in the presence of the indicated anti-β-glucan mAb (1 µg/well) or an opsonizing anti-*C. albicans* mannoprotein (MP) serum (10 µl/well) or with an irrelevant mAb or serum, at equal doses. Percent killing activity was calculated by comparison to parallel fungus cultures without macrophages. Values in figure are means+SE of triplicate determinations. Comparable results were obtained at different MOI (0.1∶1, 1∶1).

As predicted from previous work [20], a single pre-challenge treatment with the IgG mAb 2G8 significantly reduced fungal invasion of kidneys in infected animals. In contrast, parallel treatment of mice with the IgM mAb 1E12 was ineffective, as observed in three independent experiments with different *C. albicans* infecting doses (Fig. 2, panel A). A similar result was obtained in experiments measuring survival of mice treated with either mAb and challenged with a highly lethal, intravenous dose of fungal cells. In these experiments, a single injection of the IgG mAb was found to induce a slight but significant increase of survival rates and a significantly prolonged median survival times of treated animals, whereas mice receiving the IgM mAb died with rate and extent similar to saline-receiving controls (Fig. 2, B).

The two mAbs were also tested for protection in a well-established, self-healing model of rat experimental vaginitis in which animals received a “therapeutic” antibody treatment 1, 24 and 48 h post-intravaginal infection. As shown in Fig. 2, panel C, rats treated with the IgG mAb exhibited an accelerated fungal clearance from the vagina, with CFU values significantly lower than those found in control animals at all time points, and an earlier resolution of the vaginal infection (indicated by CFU-negative vaginal fluid cultures on day 28). In comparison, the IgM mAb only caused some accelerated, statistically significant, decay of the vaginal fungus burden at early time points (≤5 days), but not at later time points, and was not able to accelerate the eradication of *C. albicans* from the vagina.

The two mAbs were therefore compared for their ability to opsonize fungal cells, thus promoting their phagocytosis and killing by phagocytic cells. Opsonisation is a critical property of protective anti-*Candida* antibodies [39] and, as shown above, some β-glucan constituents, bound by both the IgG and the IgM mAb, were present on cell wall surface (Figure 1). In a CFU count-based killing assay using the murine macrophage cell line J774 (Fig. 2, panel D) both mAbs were unable to increase the anti-*Candida* activity of murine cells, in marked contrast with a positive control, an anti-*C. albicans* mannoprotein serum, which proved effectively opsonic.

Taken together, the results from protection experiments in two very different experimental models of candidiasis consistently highlighted a remarkable anti-*C. albicans* protective potential for the IgG mAb and little or no protective activity for the IgM mAb. The results also indicated that this difference in protective activity was unlikely to depend on differing opsonisation properties of the two mAbs.

### Both the IgG and the IgM mAbs recognize fungal β-glucan but they differ in fine epitope recognition

**Figure 3:**
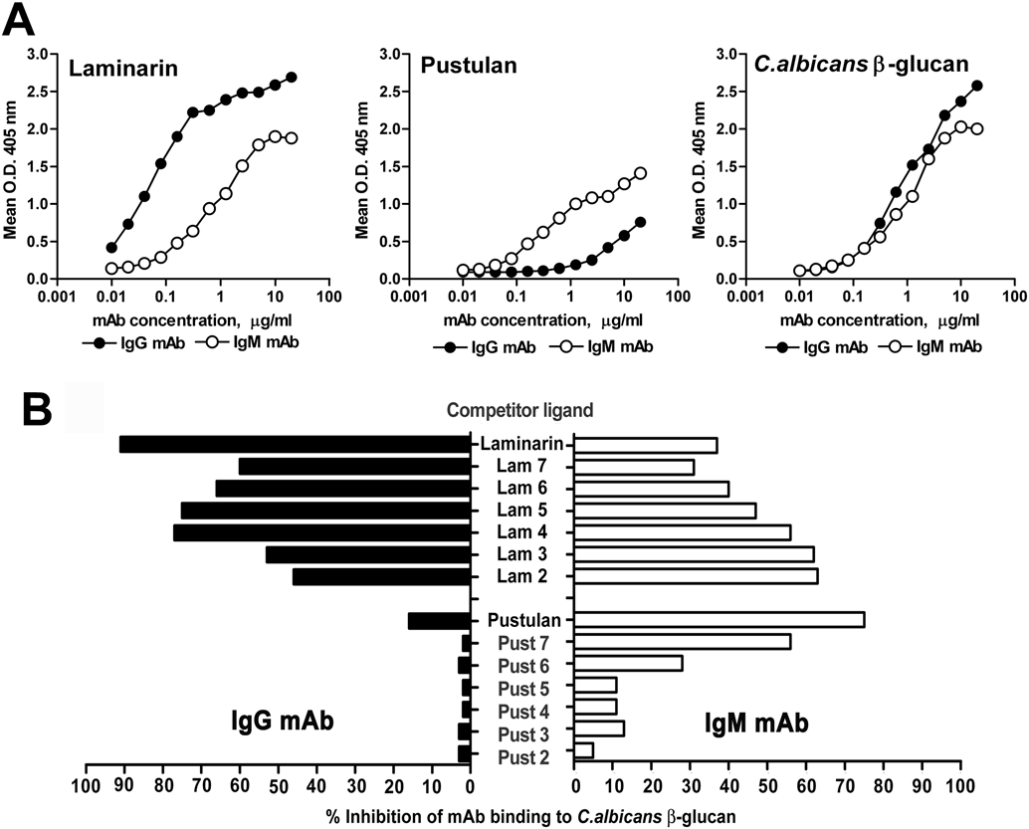
Reactivity of the antiβ-glucan mAbs to β-glucans of different molecular structure. Panels A: dose-effect, ELISA mAb binding curves to plastic-adsorbed laminarin ( β1,3 glucan), pustulan (linear β1,6 glucan) and *C. albicans* β-glucan (mixed, highly branched β1,3/β1,6-glucan). The graph illustrates the outcome in a typical experiment out of five performed with similar results. Binding is expressed as mean O.D. 405 nm readings from triplicate wells after subtraction of O.D. from the negative controls (wells reacted with no mAb or with an irrelevant mAb). SEM values were always <15% and are not shown. Panel B: ELISA competition assays. Laminarin, pustulan and two series of β1,3 or β1,6 oligosaccharides of DP 2 to 7 (Lam or Pust 2 trough 7, respectively) were tested for ability to inhibit the binding of the mAbs to plastic-adsorbed, *C. albicans* β-glucan. Competitive binding activity is expressed as percent reduction of mAb O.D. 405 nm readings in the presence of the various competitor ligands, as compared to O.D. readings in the absence of competitors. Data in the figure are those from one representative experiment, out of three performed with similar results, using the mAbs at 1 µg/ml and 10 µg/ml and 50 µg/ml of β1,3- and β1,6-linked saccharide competitors, respectively.

The data reported above invited to investigate other properties of the two mAbs that could account for their different protective capacity, including possible differences in fine antigenic recognition. This, in fact, could be modulated by the isotype-related constant region, as observed for other antibodies [40]–[44]. We started by examining any differential recognition of the two isomeric β1,3 and β1,6-glucan sequences, which are both present and intermixed in fungal β-glucans. Initial experiments were performed by ELISA and competition ELISA assays, using various β-glucan poly- and oligosaccharides. Figure 3, panel A, shows a comparison of dose-response mAb binding to laminarin, a β1,3-linked linear glucan molecule with occasional β 1,6 branches of glucose [45], pustulan, a β1,6-linked, linear glucan [46] and to soluble, purified *C. albicans* β-glucan (a highly branched glucan with mixed β1,3- and β1,6-linked components, also referred to as GG-Zym [47]). The data indicated that the IgG recognized strongly laminarin, and only weakly, at high antibody concentration, pustulan. In contrast, the IgM bound both to laminarin (though less strongly than the IgG) and pustulan. The binding of the two mAbs to *Candida* β-glucan (GG-Zym) was rather similar, in keeping with the mixed β-1,3 and β-1,6 sequences in this glucan. Curve fit analysis and comparison of slopes confirmed a significant difference between IgG and IgM binding curves to laminarin (P<0.0001) and pustulan (P = 0.003), but not to GG-Zym (Fig 3, panel A).

ELISA competition assays were performed to gain preliminary insights into specific oligosaccharide structures of fungal glucan recognized by the two antibodies. GG-Zym was used as the plastic-bound antigen and laminarin, pustulan and a number of β1,3- or β1,6-linked oligosaccharides with a degree of polymerization (DP) 2 to 7 were assayed in liquid-phase as inhibitors of the mAbs. As shown in Fig. 3, panel B, binding of the IgG mAb to GG-Zym was almost abolished in the presence of laminarin and strongly inhibited by β1,3-linked oligosaccharides, but unaffected by β1,6-linked oligosaccharides. In contrast, the binding of the IgM mAb was strongly inhibited by pustulan and, with decreasing magnitude at decreasing of DP, by β1,6-linked oligosaccharides, and also by β1,3-linked oligosaccharides, particularly by di-tri- and tetra-glucosides (Fig. 3, panel B).

**Figure 4:**
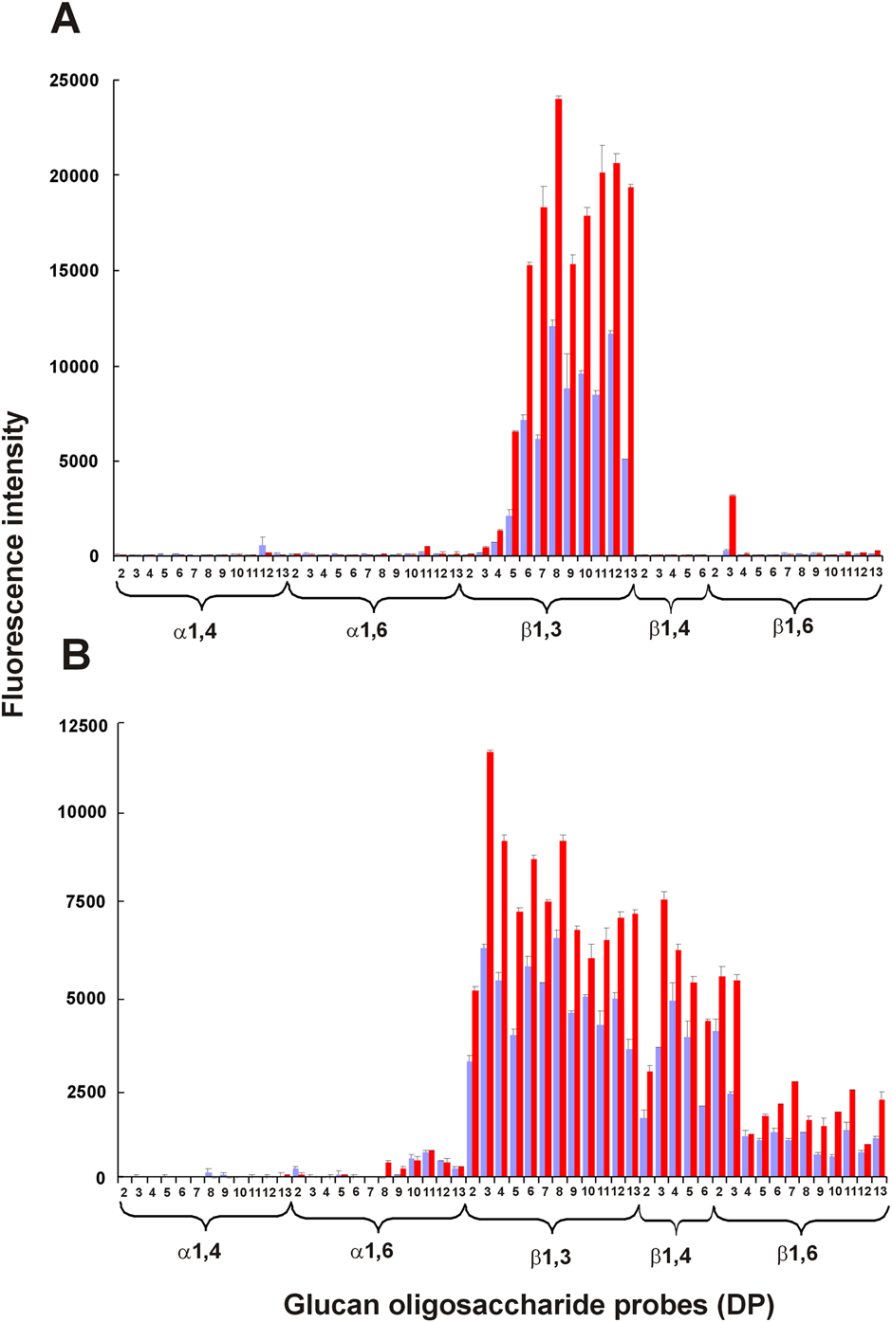
Microarray analyses of the interactions of the mAbs with different gluco-oligosaccharide probes. The gluco-oligosaccharide probes were printed as duplicate spots and the binding was assayed with the IgG mAb at 0.1 µg/ml (Panel A) and the IgM mAb at 0.5 µg/ml (Panel B). Numerical scores are shown for the binding signals, means of duplicate values at 2 and 7 fmol/spot (blue and red bars, respectively, with error bars). The gluco-oligosaccharide probes tested included oligosaccharides from maltodextrins (α1–4), dextran (α1–6); curdlan (β1–3); cellulose (β1–4); and pustulan (β1–6). Numbers on the X axis indicate degree of polymerization (DP) of the major components in the oligosaccharide fractions.

These data prompted us to carry out a more detailed characterization of the epitopes recognized by the two mAbs using microarray analyses. The arrays contained five series of linear glucan oligosaccharides of different configurations and DP (up to 13 glucose units) arrayed as neoglycolipids (Fig. 4). The IgG mAb bound to the laminarin-type oligosaccharides containing the β1,3 linkage, starting from a minimum DP of 4, and the binding strength increased with increasing oligosaccharide chain length (maximum binding to octaose, then plateauing) (Fig. 4, A). There was little or no binding to the oligosaccharides from maltodextrins (α1,4), dextran (α1,6), cellulose (β1,4) and pustulan (β1,6) except that there was unexplained moderate binding to the trisaccharide probe from pustulan. In contrast, the IgM mAb bound not only to the β1,3 oligosaccharides but also to those with β1,4 and β1,6 linkages (Fig. 4, B). Coherently with the ELISA data (Fig. 3), the specific activity of the IgM antibody toward the β1,3-linked oligosaccharides was lower than that of the IgG mAb: the signals given in Fig. 4A and B were elicited with 0.1 and 0.5 µg/ml of the IgG and IgM mAb, respectively. Taken together the ELISA inhibition and the glycoarray data showed that the IgG mAb has a greater specific activity and a more restricted specificity for the β1,3 glucan sequence than the IgM mAb.

### IgG mAb-reactive, IgM mAb unreactive cell wall and secretory proteins

**Figure 5:**
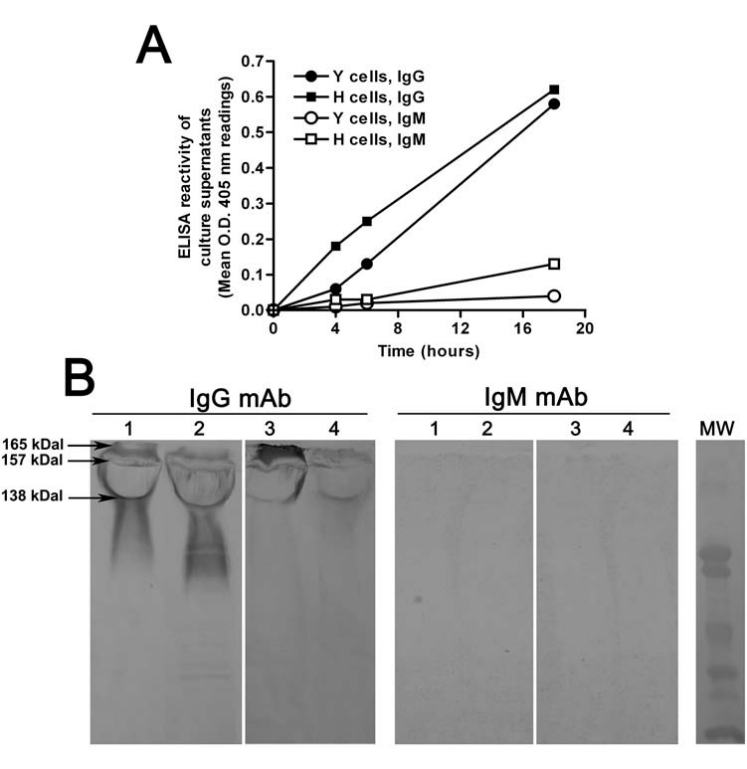
Reactivity of the anti-β-glucan mAbs to the secretory material and cell wall proteins of *C. albicans*. Panel A: Secretion of IgG- or IgM-reactive β-glucan material during fungal growth. Serial dilutions of fungal culture supernatants obtained at different times of growth in the yeast (Y) or hyphal (H) form were adsorbed (in duplicate) onto polystirene plates and reacted with the mAbs in ELISA. The figure shows reactivity of fungal supernatants diluted 1∶100, as from one representative experiment out of two performed independently. Panel B: Western blot analysis of culture supernatants and cell wall proteins. Culture supernatants (lane 1, 50 µg and lane 2, 25 µg polysaccharide) and cell wall proteins (lane 3, SDS-extracted and lane 4 β1,3-glucanase-extracted) were reacted with the anti-β-glucan mAbs, as indicated. Calculated molecular weight for IgG-reactive bands is also indicated. MW: molecular weight markers (104.3, 97.3, 50.4, 37.2, 29.2, 20.2 kDal).

Notoriously, fungal cells release abundant β-glucan during their growth in vitro and in vivo, so that β-glucan detection in patients' serum is valued as a diagnostic marker of invasive fungal infections [48]. We therefore assayed supernatants of *C. albicans* cultures for reactivity with the IgG or the IgM mAb. In ELISA experiments (Fig. 5, panel A), we observed that the material progressively released during growth by both yeasts and hyphae of *C. albicans* contained mAb-reactive β-glucan material which was, however, much more reactive with the IgG than with the IgM. This suggested that epitope conformation of the secreted β-glucan did not overlap with that of β-glucan expressed on cell surface, which was instead rather similarly reactive with the IgM as with the IgG (see Fig 1).

We considered that β-glucan is often secreted by fungi in association with cell wall proteins, in particular the mannoproteins [49], and that several cell wall proteins which are secreted into the external milieau are known to be covalently linked to β-glucan [50]–[52]. Thus, the secreted material was analyzed by SDS-PAGE and Western blot to identify possible, discrete protein components bearing mAb-reactive motifs. As shown in Fig 5 (Panel B), abundant IgG-reactive material was indeed detected in both hyphal and yeast secretion. For its highly heterogeneous and polydisperse appearance this material likely consisted mostly of molecularly ill-defined, variously sized polysaccharides. Nonetheless, a number IgG-reactive bands, in particular three bands with an approximate molecular weight of 165, 157 and 138 kilodaltons, were coarsely distinguishable within the smear. Apparently similar mAb 2G8-reactive, faint bands were also detected among cell wall proteins extracted by SDS- or β-(1,3)-glucanase treatment from isolated fungal cell wall (Fig. 5B), suggesting that the IgG-reactive, secreted proteins originated from fungal cell wall. None of the components present in the secretory material or in the cell wall protein extracts was recognized by the IgM mAb (Fig. 5B).

As expected from the abundance of mannoproteins in the culture supernatant and the sensitivity of their mannan component to periodate oxidation, the secretory material was also very reactive with Concanavalin A, a reactivity that was completely lost upon periodate treatment (Fig. S1). Oxidation also affected some IgG mAb-reactive constituents, but it left completely intact other components, inclusive of those corresponding to the 157 and 138 kDal bands (Fig. S1), in keeping with the expected resistance of β1,3 glucan to periodate oxidation.

By immuno-affinity purification onto a mAb 2G8-Protein A-Sepharose column, the IgG mAb-reactive material was isolated from culture supernatants yielding a fraction that comprised at least two of the reactive bands observed in total fungal secretion, in particular the component with an apparent molecular weight of 138 kDal (Fig. S2). Interestingly, this fraction was also recognized by sera from mice immunized with the Lam-CRM vaccine [20], suggesting that at least some of the anti-β-glucan antibodies generated by this protective vaccination have the same specificity as the protective IgG mAb (Fig. S2).

To gain insights into protein constituents associated with the IgG-reactive, secreted β-glucan, the two bands of 138 and 157 kDal, best recognizable in the fungal secretion, were excised from the gels, subjected to controlled proteolysis with trypsin and analyzed by mass spectrometry. Following this approach, the analyses of both bands yielded several peptide mass signals with signal/noise ratio (S/N) >5. A MASCOT search was carried out against the fungal protein sequences in the NCBInr database and Als3 was clearly identified as a component of both bands. Furthermore, in the 138 kDal band the search also identified the Hyr1 protein (Table 2). The majority of the signals present in the mass spectra (9/13 in the 157 kDal and 13/17 in the 138 kDal band) matched with the sequences of the protein identified.

**Table 2:** Identification of Als3 and Hyr1 proteins in fungal secretion.

Band (MW,daltons)	Sequence of identified peptides	AA position	Protein and main function
	FTTSQTSWDLTAHGVK	77–92	
	ALGTVTLPLAFNVGGTGSSVDLEDSK	124–149	
	KISINVDEFR	166–175	
**157,000**	GYLTDSR	182–188	Als3
**138,000**	GDVQIDCSNIHVGITK	221–236	Adhesin
	GLNDWNYPVSSESFSYTK	237–254	
	APFTLR	306–311	
	WTGYR	312–316	
	GGIQGFHGDVK	31–41	
	STAYLYAR	135–142	
**138,000**	LGNTILSVEPTHNFYLK	206–222	Hyr 1
	LGLTLPLTGNR	250–260	Hyphal growth
	FEYYPDTGILQLR	265–277	
	AAALPQYFK	278–286	

Overall, these results, coupled with those illustrated in the previous sections, indicated that β-glucan antigenic motifs bound by the two mAbs are expressed in the cell wall and at cell surface, and are secreted into the external milieu. However, significantly more IgG-reactive components are secreted, and these include those associated with Als3 and Hyr1, two GPI-anchored cell wall proteins that exert critical roles in cell wall assembly, growth and fungal virulence [50]–[53].

### The IgG mAb, but not the IgM mAb, has a direct fungal growth-inhibitory activity and inhibits adherence of fungal cells to mammalian epithelial cells

**Figure 6:**
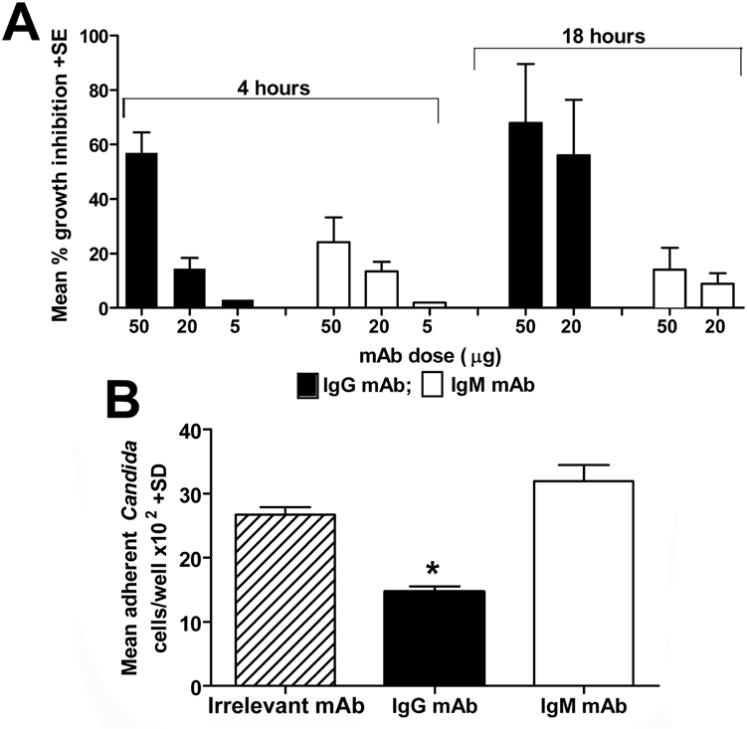
Antifungal properties of the anti-β-glucan mAbs. Panel A: *C. albicans* growth-inhibitory activity by the mAbs. *C. albicans* cells were cultured at 37°C in the presence or in the absence of the indicated mAb doses and, after 4 or 18 hours of incubation, fungal growth was estimated by a standard CFU counts. Percent growth inhibition values are from three independent experiments and were calculated by comparison to fungal cultures with equal doses of heat-inactivated mAbs or with an irrelevant mAb. Statistically significant differences between growth inhibitory activities by the IgG and the IgM mAb are marked by asterisks. Panel B: ability by the mAbs to inhibit adherence of *Candida* to human epithelial cells. Monolayers of Hep-2 cells were put into contact with fungal cells which have been pre-treated with the IgG, the IgM or with an irrelevant mAb as indicated. After a 1 h contact at 37°C, co-cultures were gently washed to eliminate non adherent fungi. Adherent fungal cells were recovered from the wells and enumerated by a standard CFU counts. Data in the graph are from three independent experiments, each performed in triplicate.

We have previously reported that the protective anti-β-glucan antibodies generated by Lam-CRM vaccination could directly inhibit fungal growth in vitro, without the contribution of any host effector cell, a property which might greatly contribute to their documented anti-*Candida* defensive action in vivo [20]. Thus, we compared the two mAbs in growth inhibition assays where *Candida* cells were treated with the mAbs for various times and then enumerated by a standard CFU count to evaluate the mAb inhibitory effect. Corroborating a preliminary report [20], the IgG mAb proved efficacious in reducing fungal growth as early as after a 4 h contact and in a dose-dependent manner. This effect became even more pronounced after 18 h of contact. By comparison, the IgM mAb showed little activity: at the highest concentration (100 µg/ml) it gave a maximum of 10–30% growth inhibition, which was well below the 70–80% afforded by the IgG at the same concentration (Figure 6, panel A).

We also tested the two mAbs for any effects on adherence of *C. albicans* hyphae to monolayers of Hep-2 cells. Following preliminary dose-response experiments (not shown), the best effective concentration of 100 µg/ml of each mAb was selected. As shown in Figure 6, panel B, approximately 45% fewer adherent hyphae were counted in the presence of the IgG mAb, as compared to control epithelial cells-*C. albicans* co-cultures with an irrelevant mAb (P<0.001). The IgM mAb, here again, was devoid of any inhibitory effect.

## Discussion

The salient findings in this study are: *i*) an IgG and an IgM anti-β-glucan monoclonal antibody (mAb) that bound to the cell surface of a number of pathogenic fungi and shared identical CDRs, differed in their specificity toward β-glucan sequences; *ii*) protective activity in experimental animal models of *C. albicans* infection was shown by the IgG mAb, which selectively recognized β1,3 glucan rather than by the IgM, which was promiscuous in its binding to β1,3, β1,6 and also β1,4 glucan sequences; *iii*) the protective IgG mAb but not the non-protective IgM mAb, bound to components secreted by growing fungal cells; *iv*) the IgG mAb caused a remarkable inhibition of *C. albicans* adherence to epithelial cells and reduced hyphal growth *in vitro*; *v*) the IgG and also the IgM were not opsonic in a macrophage opsonisation-killing assay, but the IgG was able to bind to at least two glucan-linked cell wall proteins, the Als3 and Hyr1, which play a role in adherence, tissue-invasion and growth of *C. albicans* [54], [55]. The last two findings suggest that the protective efficacy may be at least in part mediated by host-independent properties.

A number of experimental approaches were used to gain insights into the cell wall ligand(s) recognized by the two mAb. These consistently indicated that the protective IgG mAb had a quite selective specificity for β1,3- linked glucose sequences. Its binding to fungal β-glucan was inhibited by β1,3- but not by β1,6-linked oligosaccharides in competition ELISA experiments. Microarray analyses showed exclusive IgG mAb binding to β1,3-linked glucose sequences with DP 4 and greater. In contrast, the IgM mAb bound both to β1,3- (although less strongly than the IgG mAb) as well as to β1,6-linked glucose sequences with DP 2 and greater. As shown by microarray analyses it also recognized saccharides with β1,4 glucan sequences, which are ordinarily absent in fungi but present in higher plants and lichens. Interestingly, a linear β1,3-β1,4-linked glucan is uniquely present in the cell wall of *Aspergillus fumigatus* [56], a fungus which is bound by both mAbs, as found by immunofluorescence staining, and whose growth is highly susceptible to the anti-β-glucan antibodies generated by the immunization with the Lam-CRM vaccine [20].

The disposition of β1,3 glucan on *C. albicans* cell surface, an essential prerequisite for interaction with the protective anti-β-glucan mAb, is a matter of controversy. There are suggestions that β1,3 glucan is hidden inside the cell wall by a layer of mannoprotein constituents and that it is lacking or negligibly expressed on the fungal surface, particularly in hyphal cells [57]–[60]. This sort of β-glucan stealth has been recently interpreted as a host-deceiving mechanism, as it would prevent the binding of fungal cells by Dectin-1 - a β1,3 glucan specific receptor present on a variety of cells of the innate immune system - thus inhibiting the triggering of critical signals for coupling innate to adaptive antifungal immunity [57], [59]–[63]. However, other authors have reported surface expression of this polysaccharide in both yeasts and hyphae [20], [64]. This apparent discrepancy could be reconciled by very recent reports that β1,3 glucan may be variably expressed by fungal cells depending on their growth conditions and/or morphology [65], [66]. Moreover, different reagents, with likely different fine specificity, were used by the various Authors to detect β1,3 glucan at cell surface. The IF and EM data shown here with the use of a mAb with restricted specificity for β1,3 glucan leave little doubt that some β1,3 glucan or components carrying this polysaccharide are expressed on cell surface, albeit not in all cells of the culture ( especially for yeast cells) and not uniformly in all labelled cells (especially for hyphal cells). This would be expected in a asynchronous culture if β1,3 glucan is consistently exposed only at certain stages of growth. Interpretation of all these data must take also into account the presence of β1,3 and β1,6 glucan moieties in the mannoprotein modules of the cell wall, which are both intrinsic cell wall constituents and secretory molecules somewhat spanning the cell wall and transiently expressed on cell surface [67]. In this scenario, the antibody-accessible β-1,3 glucan could be mostly represented by the glucan bound to the proteins of the outer cell wall, which is likely to undergo even marked variations during growth and morphologic changes, rather than the more invariant β-glucan of the inner cell wall skeleton.

In this paper, we show that there is at least as much IgM- as IgG mAb-reactive β-glucan in *C. albicans* cell wall, as seen by ELISA using *C. albicans* β-glucan (GG-Zym; see above) and EM of gold-immunolabelled sections of both yeast and hyphal cells of the fungus. Remarkably, however, the material secreted by growing fungal cells was much more reactive with the IgG than with the IgM mAb (in ELISA) or substantially non reactive with the IgM antibody (in Western blot). This suggests that the secreted material is enriched with β1,3 glucan sequences as compared with β-glucan “stably” located in the cell wall.

Among the secretory components recognized by the protective IgG mAb, two of them, Als3 and Hyr1, could be among the targets of the protective action of this mAb. Both these proteins belong to a major category of cell wall components, the GPI-anchored proteins, which are covalently linked to β-glucan and include structural constituents, enzymes, adhesins and other components playing a crucial role in cell wall organization, stress response and virulence of the fungus [50], [68]. Both Als3 and Hyr1 are known to be actively secreted by *C. albicans* [50], [68]: their strong binding by the IgG mAb would also suggest that they are secreted with attached β-glucan, in particular β1,3 glucan, moieties.

Relatively little is known about the functional properties of Hyr1 protein. It is activated during hyphal transition [69] and upon exposure to macrophages or neutrophils [70], [71] and its expression has been shown to be under control of well-established hyphal regulatory pathways of this fungus [72], [73]. Overall, this protein is assumed to play a role in yeast to mycelial conversion of *C. albicans*. Relevant to the data shown in this study is a very recent report that members of the IFF protein family, to which Hyr1 belongs, play a role in fungus adherence [74].

There is much more information on the structure and biological properties of Als3, and this is clearly relevant to the interpretation of the inhibitory and protective potential of our anti-β1,3-glucan IgG mAb. Als3 is encoded by the corresponding gene of the *ALS* gene family which code for structurally-related, high Mr cell surface and secreted glycoproteins [75]. Their primary, though not exclusive, biological role is to mediate adherence of fungal cells, possibly with different members of the family being differentially involved in the adherence to different host cells and tissues [75]. As pointed out by results of gene overexpression, knock-out mutant studies in *C. albicans* and heterologous expression in *S. cerevisiae*, Als3 is the member of the family with the largest impact on adherence to both epithelial and endothelial cells, [76]–[78]. Together with other adhesins, Als3 is also involved in biofilm formation [79]. The Als3 protein has also been shown to mediate iron acquisition from ferritin by the hyphae of *C. albicans* [80], being iron acquisition necessary for hyphal growth.

Recombinant Als3 and its N-terminus moiety have been quite extensively investigated as candidate vaccines against *C. albicans* and other *Candida* species, and shown to be protective both against systemic and mucosal candidiasis [37]. Vaccine efficacy has been postulated to be entirely dependent on cell-mediated immunity, with little or no role for antibodies [37]. In its recombinant format, the Als3 protein lacks β1,3 glucan, thus any role of anti-β1,3-glucan antibodies in the above context is excluded. Nonetheless, Als3 is an adhesin/invasin with multiple roles in virulence and this would suggest that anti-Als3 antibodies directed against the native Als3 could exert protection by blockade of one or more virulence-associated epitopes of the protein. Of interest in this context is that another protective antibody (mAb C7), which is directly candidacidal, has been reported to bind to the N-terminus region of Als 3 [81].

Overall, both Hyr1 and, more evidently, Als3 play important roles in *C. albicans* virulence properties such as hyphal growth and adherence which are both inhibited by our protective IgG mAb which recognizes the two proteins. In contrast, neither hyphal growth nor adherence are affected by the non protective IgM mAb which does not recognize the two proteins. Nonetheless, it remains possible that the protective antibody interacts with, and inhibits the function of other unidentified β1,3 glucan constituents exerting a role in fungal virulence or other critical biological properties *in vivo*.

It is rather surprising that neither the Hyr1 nor the Als3 proteins are bound by the promiscuous IgM mAb which recognizes different β-linked saccharide molecules, including β1,3 glucose sequences. However, it should be considered that mAb epitope specificity data shown in this study have been obtained using polysaccharides, free oligosaccharides and lipid-linked oligosaccharide probes. It is possible that the β-glucan antigen on native and secreted proteins is presented in a form that can be bound by the IgG but not by the IgM. In principle, the low specific activity of the IgM mAb for the β1,3 glucan, as assessed with laminarin or oligosaccharides used in ELISA and microarrays, could be even lower for the β1,3 glucan sequences of the secreted native proteins. Extensive mannosylation of the two proteins could also exert steric hindrance toward the access of the large IgM to the β-glucan epitope.

As outlined above, the two mAbs used throughout this study have different isotypes but identical amino acid sequence of both light and heavy chain CDRs. A few examples of mAbs with the same CDR but different isotypes were reported to have different binding specificities and avidities toward polysaccharides, because of the influence exerted on binding by structural elements of the antibody distant from the antigen-binding site [41], [42], [44], [82]. These factors may also influence the biological properties of the antibody, including its protective ability against infections [38], [40]. It is well known that antibody isotype influences the avidity whereby the antibody binds its cognate antigen, as well as a number of Fc-dependent, host effector functions, such as, for instance, complement activation and interaction with activating or inhibitory receptors on various immune cells. However, other mechanisms translating fine differences in antigen binding of antibodies sharing the same CDR into profound differences in their biological properties have not been established. We show here for the first time that the isotype contributes to the selectivity and intensity of binding to particular, structurally defined, β-glucan sequences as well as to defined cell wall molecules of the fungus. As an important consequence, we show here that antibodies with different isotypes may differ in their ability to inhibit critical virulence properties of a fungal pathogen such as hyphal growth and adherence *in vitro*.

It remains to be determined whether the isotype-dependent protective properties of the IgG mAb are also independent of Fc-mediated antibody effector mechanisms *in vivo*. Opsonisation is one of the main mechanisms whereby the host can eliminate or control *Candida* in vivo [40]. Our anti-β-glucan IgG mAb did not show opsonic potential in the macrophage model system tested. The lack of opsonisation appears to be in keeping with the discontinuous nature of β-glucan expression at the fungal cell surface and may be relevant also to the interactions with other effector systems (e.g. those mediated by neutrophils) not addressed here. Nonetheless, other Fc-dependent and independent biological activities of this antibody could play a role in *vivo*, and these need to be evaluated in future studies. For instance, the protective mAb, through its binding to the secreted β1,3-glucan and Als3 could inhibit biofilm formation to which both this polysaccharide and the ALS3 protein seem to play a role [79], [83], and thus interfere with this process which has a key role in fungal invasion. Conceivably, this antibody may also modulate, to the host's advantage, the interactions of fungal cells with Dectin-1 and other critical receptors of innate immunity [84] or also abrogate the inhibitory capacity expressed by some β-glucans on maturation of host dendritic cells, which are critically involved in the generation of protective anti-fungal immunity [85], [86].

Taken together, the data presented in this study identify blockade of adherence and interference with hyphal growth as possible mechanisms of protection by anti-β-1,3-glucan antibodies. This highlights the exciting possibility that antibodies which neutralize virulence factors of the fungus, thus not relying entirely upon host factors for their therapeutic activity, would be of value in the fight against pathogenic fungi in immuno-compromized subjects. Nonetheless, further studies are needed to address in detail the mechanisms of antibody protection *in vivo*.

## Materials and Methods

### MAb generation and characterization

2G8 and 1E12 hybridomas were generated from spleen cells of Balb/c mice (Harlan Nossan, Indianapolis, Indiana) previously immunized with a soluble, *C. albicans* β-glucan preparation (GG-Zym, [35] conjugated to the recombinant, genetically inactivated diphteria toxin CRM_197_, as already described [20]. Spleen cells were fused with myeloma cells of the murine line X63-Ag8 653 using standard in-house protocols [87]. Hybridomas were selected by assaying the secreted mAbs by ELISA with a panel of β-glucan molecules (laminarin, pustulan, soluble *C. albicans* β-glucan ); isotype (IgG2b and IgM for 2G8 or 1E12, respectively) was determined by reactivity with specific, alkaline-phosphatase-conjugated anti-mouse immunoglobulin antibodies. Hybridoma cells were routinely cultured in protein-free CD Hybridoma medium (Gibco, Grand Island, NY, USA), supplemented with 100 U penicillin/ml, 100 µg streptomycin/ml, 1 mM sodium pyruvate, and 2 mM L-glutamine (Hyclone, Logan, Utah). MAbs were precipitated from culture supernatants by ammonium sulfate [87], dyalized against PBS and concentrated by ultrafiltration through a 100 kDa cut-off membrane (Millipore, Bedford, USA) and measured by a commercial protein assay (BioRad, Richmond, USA) following the manufacturer's instructions. The irrelevant, negative control mAb (IgG2b immunoglobulins directed against unconjugated CRM_197_ protein) was obtained from the respective hybridoma following an identical procedure. In some growth inhibition assays, heat-inactivated (5 min, 100°C), anti-β-glucan mAbs were also used as the negative controls.

For sequencing VH and VL variable regions of the mAbs, mRNA was isolated from approximately 1×10^7^ hybridoma cells, using QuickPrep *Micro* mRNA Purification Kit (# 27-9255-01, Amersham Pharmacia Biotec, HP7 9NA Buckinghamshire, UK), according to the manufacturer's instructions and reverse-transcribed (1 µg from each hybridoma cell line) by a Smart PCR Synthesis kit (# K1052-1, Clontech, USA). cDNA quality was analysed by polymerase chain reaction (PCR) using specific primers to amplify GAPDH as housekeeping gene. L chains were amplified by PCR with the VL1 fw *5′-GATATTGTGATGACCCAGTCTCCA-3′* and VL2 Rev *5′-TGGATACAGTTGGTGCAGC-3′* primer, whereas H chains were amplified with VH1 BACK *5′-AGGTSMARCTGCAGSAGTCWGG-3′* and VH2 *5′-GGCCAGTGGATAGAC-3′* . The amplified VH and VL chain DNA samples were separated on a 2% agarose gel, purified and cloned into pCR-bluntII-TOPO (Invitrogen, CA 92008 USA). The *Escherichia coli* TOP 10 strain was used for transformation experiments with recombinant plasmids, according to Zero Blunt TOPO PCR Cloning Kit methods (# K 2800-20, Invitrogen). Transformant clones were analysed by PCR on colony, and amplified fragments were digested with HaeIII restriction endonuclease. All constructs were sequenced by Biofab Research srl, (Rome, Italy), and VH and VL CDRs of the hybridoma cell lines were analysed with the Imgt database (http://imgt.cines.fr/). All procedures described above were performed twice and identical VH and VL gene sequences were obtained. Gene sequences were submitted the GenBank and received the accession number of the nucleotide sequence : FJ790243 (bankit1189566).

### Microorganisms

*C. albicans* strains BP and SA-40 (type collection of the Istituto Superiore di Sanità) were used in the models of disseminated and vaginal *Candida* infections, respectively. For experimental infections, cells from stock cultures in Sabouraud-dextrose agar (Difco-BBL, Franklin Lakes, New York) were grown in Winge medium (strain BP, [35]) or in YEPD medium (1% yeast extract, 2% peptone, 2% glucose, all w/v, strain SA-40, [29]) at 28°C for 24 h, then harvested by centrifugation, washed, counted in a hemocytometer, and resuspended to the desired concentration in phosphate-buffered saline (PBS). Yeast cells for in vitro experiments were prepared as described above using the strain BP. Fungal hyphae were obtained by culturing yeast cells at 37°C in Lee's medium or in RPMI 1640 (Euroclone Ltd.), supplemented with 1 mM glutamine and 2% FCS (EuroClone), as previously described [88]. Under these conditions, initial yeast cells developed short and elongated germ-tubes within 2–6 h, respectively, and hyphal filaments at 18 h. In some experiments, parallel, yeast-form cultures were grown under the same conditions at 28°C. *A. fumigatus* 495 and *C. neoformans* ATCCL, from the type collection above, were routinely maintained on Sabouraud-dextrose agar slants. *A. fumigatus* hyphae were grown from conidial suspensions as described in [20]. *C. neoformans* yeast cells were grown for 18 h in Sabouraud-dextrose broth under slight agitation, washed and resuspended in PBS.

### Immunofluorescence and immunoelectron microscopy

For immunofluorescence staining, live yeast or hyphal cells of *C. albicans* or *C. neoformans* cells, were allowed to adhere on immunofluorescence microscope slides, extensively washed with PBS containing 0.1% Tween 80 and blocked (1 h, 37°C) with 3% bovine serum albumin (BSA) in PBS. Spots were reacted (2 h, 37°C) with various dilutions of the mAbs in PBS-3% BSA, washed, and treated with fluorescein isothiocyanate- (FITC)-conjugated-anti mouse IgG or IgM antibodies (Sigma-Aldrich, 1 h, 37°C). After extensive washings, the slides were mounted in glycerol, pH 9.6, and examined under a Leitz Diaplan fluorescence microscope. *A. fumigatus* hyphae were prepared by culturing conidia (10^4^ in RPMI 1640-FCS) in microscope chamber slides (NUNC, Roskilde, Denmark). After incubation for 18 h at 37°C, the culture medium was removed, the slides were washed, blocked, reacted with the mAbs and observed as described above. Parallel staining with negative control mAbs or with the secondary antibodies alone was carried out in all the experiments. Ultrathin cryosections of *C. albicans* yeast and hyphal cells were prepared following the method by K.T. Tokuyasu [89]. The cells were fixed with 4% (w/v) paraformaldehyde plus 0.5% (v/v) glutaraldehyde and embedded in 2% (w/v) agarose low melting point (LMP). Agarose blocks were infused with 2.3 M sucrose and frozen in liquid N_2_. Ultrathin cryosections obtained by Leica Ultracut UCT device (Leica Microsystem, Wien, Austria), were incubated with the mAbs, then revealed with specific goat anti-mouse 10-nm gold conjugates (1∶10 diluted, Sigma-Aldrich, Milan, Italy). Samples were examined with a Philips 208 transmission electron microscope (FEI Company, Eindhoven, The Netherlands). Negative controls were performed by reacting samples with an irrelevant murine IgG or with the immunoconjugates alone.

A quantitative evaluation of the labeling intensity was carried out on micrographs of cryosectioned hyphal and yeast forms, after labeling with IgG or IgM antibodies. Six images for each sample were chosen, paying particular attention to select only those in which the section crossed the central plane of the cell. In this way, the two different fungal forms could be well identified and the thickness of the cell wall was easily measurable. The gold particles present on the section plane of the cell wall were counted manually and related to the respectice surface (µm^2^).

### Protection assays

Protection against experimental, systemic candidiasis was assessed in female, 4 week-old CD2F1 mice (Harlan-Nossan, Milano, Italy). Animals were administered a single i.p. dose of either mAb (100 to 150 µg/0.5 ml, as specified in single experiments) and, 2 h later, received a systemic challenge with *C. albicans* (5×10^5^ or 10^6^ cells/0.2 ml, i.v.). Control animals were treated with the same doses of an irrelevant, anti-CRM IgG2b mAb. Extent of kidney invasion was evaluated 2 d after the challenge by enumeration of fungal CFU in the left kidney [20]. When protection endpoint was mortality, mice were followed up for 60 d to assess median survival time and ratio of dead/total challenged mice.

Vaginal candidiasis was induced in oophorectomized Wistar rats (Charles River Breeding Laboratories, Calco, Italy) maintained under pseudoestrus by the s.c. administration of estradiol benzoate (Amsa Farmaceutici srl, Rome, Italy), essentially as described previously [29]. Six days after the first estradiol dose, the animals were inoculated intravaginally with 10^7^ yeast cells in 0.1 ml of saline and then treated by the intravaginal route at 1, 24 and 48 hours post-infection with 40 µg/200 µl of the anti-β-glucan mAbs, or with the same dose of an irrelevant mAb or with 200 µl of PBS. Protection was evaluated through the estimation of fungal CFU in vagina until day 21–28 after infection, as described in previous reports [29]. All animal studies were approved by the Istituto Superiore di Sanità intramural Institutional Review Committee.

### Glucan antigens and other reagents

Soluble *C. albicans* β-glucan (GG-Zym), a mixture of β1, 3 and β1, 6 glucan, was obtained by limited β1, 3 glucanase (Zymoliase 100T, Seikagaku Corporation) digestion of particulate glucan ghosts of *C. albicans*, as previously described [47]. Laminarin, a linear β1,3-linked glucan backbone with occasional β1,6-linked branching, was purchased from Sigma-Aldrich (St Louis, Missouri) and pustulan, a β1,6–linked, linear glucan, was obtained from Calbiochem (La Jolla, California). The β1,3-linked oligosaccharides used in competition ELISA (laminaritriose to laminariheptaose) were from Seikagaku, Tokyo, Japan, whereas β1, 6-linked oligosaccharides were obtained by limited digestion of pustulan with endo-β1,6-glucanase (Prozyme, San Leandro, California), followed by size separation by chromatography on a Bio-Gel P2 extra-fine resin (BioRad), as reported in [47]. A β-glucan preparation from *Saccharomyces cerevisiae*, linear β1,3,1,4 glucan from barley and dextran, an α1,6-linked polysaccharide, all from Sigma-Aldrich, were also used. A mannoprotein preparation, comprising major immunodominat fungal antigens, was extracted and purified from *C. albicans* as previously described [90].

### ELISA and competition ELISA

Polystyrene microtiter plates (MaxiSorp; NUNC) coated with the various β-glucan antigens and blocked with 3% bovine serum albumin (Fraction V, Sigma-Aldrich) in PBS were reacted with two-fold dilutions of the mAbs followed by alkaline phosphatase-conjugated, secondary antibody reagents (anti-mouse IgG or IgM antibodies, Sigma-Aldrich), as described in previous reports [35]. Plates were then developed with p-nitrophenyl phosphate disodium (Sigma-Aldrich) as the enzyme substrate and read for absorbance at 405 nm. Readings from negative controls (wells non-reacted with the mAbs or reacted with irrelevant mAbs) were subtracted from all absorbance values.

For ELISA competition assays, the mAbs were pre-reacted at 4°C with different competitor β-glucan molecules, at various concentrations. The mAb-competitor mixtures were then added to wells with plastic-adsorbed *Candida* β-glucan (GG-Zym) and residual mAb binding to this latter was measured as described above. Percentage of inhibition values were calculated by comparing O.D. measurements from the wells with the different competitor ligands with those from wells reacted with the mAbs in the absence of competitors [87].

### Oligosaccharide probes and microarray analyses

A total of 53 gluco-oligosaccharides with differing linkages and chain lengths were arrayed as lipid-linked probes prepared from reducing oligosaccharides by oxime ligation with an aminooxy (AO) functionalized phospholipid [91]. The oligosaccharides investigated were as follows: i) α1,4-linked oligosaccharides, DP 2 to 7 (Sigma-Aldrich) and fragments, DP 8 to 13, separated by gel filtration chromatography [62] from a maltodextrins acid hydrolysate (V-labs purchased via Dextra); ii) α1,6-linked oligosaccharide fragments, DP 2 to 13, were generated from dextran (Amersham-Pharmacia, Uppsala, Sweden) by acid hydrolysis followed by gel filtration chromatography; iii) β1,3-linked oligosaccharides, DP 2 to 4 from Dextra (Reading, UK), DP 5 to 6 from Megazyme (Wicklow, Ireland), DP 7 from Seikagaku America (Falmouth, MA) and fragments, DP 8 to 13, separated by gel filtration chromatography from a curdlan acid hydrolysate (Megazyme (Wicklow, Ireland); iv) β1,4-linked oligosaccharides, DP 2 (Sigma-Aldrich) and fragments, DP 3 to 6, separated by gel filtration chromatography from a cellooligosaccharide mixture (V-labs purchased via Dextra); v) β1,6-linked oligosaccharides fragments, DP 2 to 13, were generated from pustulan (Calbiochem) by acetolysis [62] followed by gel filtration chromatography. Molecular masses of the main components of oligosaccharide fractions from gel filtration were corroborated by MALDI-MS [62].

NGLs were arrayed robotically, and the microarray analyses with anti-glucan antibodies were performed essentially as described [62]. In brief, the microarray slides were overlaid with the IgG or the IgM diluted in casein (Pierce, Illinois, US) containing 1% (w/v) BSA (Sigma-Aldrich) and 10 mM CaCl_2_, to give a final concentration of 0.1 and 0.5 µg/ml, respectively. Binding of mAb 2G8 was detected with biotinylated anti-mouse-IgG, 1∶200, and of mAb 1E12 with biotinylated anti-mouse immunoglobulins, 1∶1000, (both from Sigma-Aldrich) followed by Alexa Fluor-647-labelled streptavidin (Molecular Probes) at 1 µg/ml. Imaging was as described [62] and data analysis was performed with a dedicated software (Mark P. Stoll of the Glycosciences Laboratory, unpublished).

### Preparation and analysis of *C. albicans* cell wall proteins and secretory material

Isolated, clean hyphal cell walls were prepared by mechanical breakage of cells with 0.45-mm glass beads followed by extensive washes in cold water, as previously described [92]. Observations under an electron microscope confirmed the purity of the cell walls. Glucan-associated proteins (GAPs) were obtained by extracting cell walls with sodium dodecyl sulfate (SDS)-DTT-EDTA, followed by β1,3 endoglucanase (Zymoliase 100T, Seikagaku) digestion according to Kapteyn et al [49]. Secreted fungal proteins were separated and concentrated from supernatants of 24-h hyphal cultures by extensive ultrafiltration/dialysis through a low-adsorbance ultrafiltration membrane (molecular cut-off 10 kDa, Millipore, Bedford, MA), as described elsewhere [92]. The IgG mAb-reactive fraction of fungal secretion was purified by affinity binding to a column prepared by covalently coupling the IgG mAb to protein A-Sepharose CL4B (Pharmacia) with dimethylpimelimidate (Sigma). The bound material was eluted with 100 mM triethylamine, pH 11.5, neutralized with 2 M Tris and, after dialysis against PBS and concentration by ultrafiltration, was kept at −20°C. Comparative assessment of overall reactivity of secretory material with the mAbs was made by ELISA, as described above, by using different dilutions of fungal secretions as plastic-bound antigens.

Sodium dodecyl-sulfate polyacrylamide gel electrophoresis (SDS-PAGE) separation of GAPs and secretory proteins was performed in 5 to 15% polyacrylamide slab gels, as reported elsewhere [93]. After electrophoresis, proteins were either Coomassie-stained or electrophoretically transferred onto nitrocellulose (0.2-mm pore size), immunostained with the mAbs and revealed with alkaline phosphatase-conjugated anti-mouse IgG or IgM antibodies (Sigma-Aldrich), followed by 5-bromo-4-chloro-3-indolyl-phosphate and 4-nitroblue tetrazolium chloride solution as the enzyme substrate [93]. In some experiments, fungal secretions were subjected to periodate oxidation (30 min in 0.2 M NaIO_4_, Sigma-Aldrich), followed by extensive dialysis by gel filtration and SDS-PAGE and western blot analysis. Mannosylation of fungal secretion was assessed by staining blots with Concanavalin A-digoxigenin and alkaline phosphatase-conjugated anti-digoxigenin antibody (Boehringer-Mannheim)

### Identification of mAb-reactive fungal proteins

Protein bands were manually excised from gel and proteolysis was achieved using the In-gel Digest96 Kit™ (Millipore, Bedford, MA, USA) with 15 µl of the trypsin provided by the kit manufacturer (about 11 µg/ml in 25 mM ammonium bicarbonate) at 37°C for 3 h. After digestion, in gel tryptic peptides re-suspension was performed by incubation of each gel piece in 130 µl of a 0.2% trifluoroacetic acid (TFA) aqueous solution for 30 min at room temperature. Finally tryptic peptides were eluted from the microcolumns to a MALDI target plate with 1.3 µl of a solution of α-cyano-4-hydroxy-*trans*-cinnamic acid matrix (2 mg/ml) in 70% acetonitrile containing 0.1% TFA. MALDI-TOF analyses were performed in a Voyager-DE™ STR instrument (Applied Biosystem, Framingham, MA, USA) equipped with a 337 nm nitrogen laser and operating in reflector mode. Mass data were obtained by accumulating several spectra from laser shots with an accelerating voltage of 20,000 V. All mass spectra were externally calibrated using a standard peptide mixture containing des-Arg-Bradykinin (904.4681), angiotensin I (1296.6853), 1–17 (2093.0867) and 18–39 (2465.1989) adrenocorticotropic hormon fragments. Two tryptic autolytic peptides were also used for the internal calibration (*m/z* 842.5100 and 2807.3145).

A monoisotopic mass list from each protein spot was obtained from the MALDI-TOF data after exclusion of contaminant mass values, corresponding to those expected from porcine trypsin and human keratins, automatically achieved by the PeakErazor program (http://www.protein.sdu.dk/gpmaw/Help/PeakErazor/peakerazor.html). These peptide mass fingerprints were used to search for protein candidates in fungi protein database at the NCBI using the MASCOT software program (www.matrixscience.com) according to these parameters: one missed cleavage permission and 50 ppm measurement tolerance. Oxidation at methionine (variable modification) residues was also considered and positive identifications were accepted when at least five matching peptides masses were identified.

### MAb functional assays

Opsonic activity and inhibition of fungal adhesion by the mAbs were evaluated using the J774 murine macrophage (MΦ) and the Hep-2 human epithelial cell lines, respectively. Cells were routinely maintained in RPMI 1640 medium (HyClone) containing 10% fetal calf serum, 100 U/ml penicillin, 100 U/ml streptomycin, and 2 mM glutamine at 37°C and 5% CO_2_. For experimental purposes, cells were prepared for subculture with trypsin-EDTA (Gibco, BRL), washed, resuspended in medium as above at the desired cell concentration, transferred in 96-well microtiter plates and used 24 h later.

In macrophage (MΦ) killing assays for assessment of opsonic activity, J774 cells (2×10^4^/100 µl/well) were infected with *C. albicans* cells (2×10^4^, 4×10^3^ or 2×10^3^/100 µl/well) in the presence or absence of the mAbs (10 µg/ml) or with the same dose of an irrelevant mAb. A highly opsonic anti-*Candida* mannoprotein and an irrelevant murine serum (both used at 10 µl/well) were also included in the experiments. Control cultures consisted of *Candida* cells with or without the various mAbs or sera, in the absence of MΦ. Each condition was assayed in triplicate. After a 3 h contact, MΦ were lysed by adding 0.2% (v/v) Triton X-100 (Sigma-Aldrich) and live fungal cells were enumerated by a conventional CFU count. Percentage of killing was calculated by comparison of CFU values macrophage-fungal cell co-cultures with those from parallel cultures without macrophages.

In adhesion assays, Hep-2 cells monolayers (1.5×10^5^ cells/well) were washed with PBS and incubated 2 h at 37°C in PBS with *Candida*cells (8×10^4^/well), with or without the anti-β-glucan or control mAbs (5 to 25 µg/well), in triplicate. Non adherent fungal cells were then removed with repeated, gentle washings with PBS. Adherent fungi were recovered from cell monolayers with PBS containing 0.2% Triton X-100 and enumerated by CFU counts. Percentage of inhibition of fungal adhesion was calculated by comparing the number of adherent fungi in wells containing the anti-β-glucan mAbs with that in wells containing equal concentrations of irrelevant mAbs.

Growth inhibition activity was measured at 4 or 18 h by CFU assays in *C. albicans* cultures (1.5×10^3^/ml in 200 µl of RPMI-FCS) grown in the presence of the IgG, the IgM mAb or the control mAbs (irrelevant IgG2b mAb or heat-inactivated anti-β.glucan mAbs), as reported elsewhere [20].

### Statistical analysis

Data were analyzed by the GraphPad Prism 4 software (GraphPad Inc.). ELISA data were assessed for statistical significance by curve fit analysis. Differences in median survival time and in survival rates in *C. albicans*–challenged mice were analyzed by nonparametric two-tailed Mann-Whitney U test or Fisher's exact test, respectively. Differences in survival curves were assessed by the log-rank test. Data from CFU counts, in both in vitro and in vivo experiments, were analyzed by two-tailed Student's *t* test. Multiple comparisons were made by analysis of variance (one-way ANOVA) followed by Newman-Keuls post-test.

## Supporting Information

Figure S1 Concanavalin A- and IgG mAb-staining of hyphal secretion before and after periodate oxidation. Fungal secretions were treated 30 min with 0.1 M sodium periodate, dyalised by gel filtration and then compared to untreated secretion by SDS-PAGE and Western blot, followed by specific mannoprotein staining with digoxigenin-labelled Concanavalin A (Con-A) or the IgG anti-β1,3-glucan mAb. Lanes 1 and 3: untreated fungal secretion; lanes 2 and 4: periodate-oxidised secretion. Samples loaded onto the gel correspond to 25 µg polysaccharide.
(0.71 MB TIF)

Figure S2 SDS-PAGE and Western blot analysis of IgG mAb-reactive material purified by immunoaffinity from hyphal secretion. Lane 1 and 2 show silver staining of the total hyphal secretion and the IgG mAb-immunopurified fraction, respectively. Lane 3 and 4 shows Western blot reactivity of the immunopurified fraction with the IgG mAb (Lane 3) or with the IgG fraction of a protective serum from mice immunized with the Lam-CRM vaccine (see text).
(3.72 MB TIF)
