# Supplementary figures and images for: Protection by Anti-β-Glucan Antibodies Is Associated with Restricted β-1,3 Glucan Binding Specificity and Inhibition of Fungal Growth and Adherence
Source: PLoS One. 2009 Apr 28;4(4):e5392. doi: 10.1371/journal.pone.0005392 (PMC2670538; doi:10.1371/journal.pone.0005392)

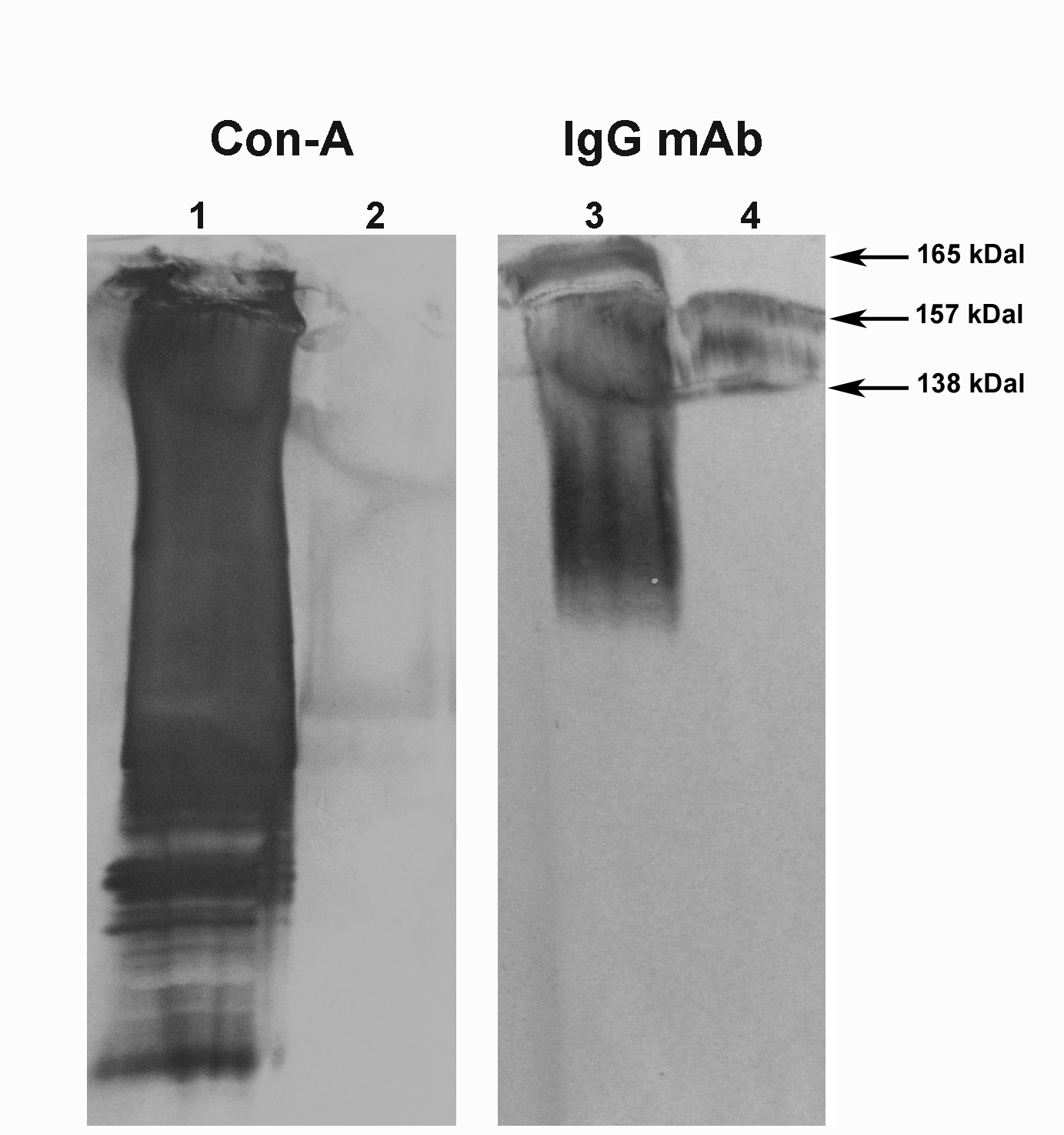

Supplement: Figure S1 — Concanavalin A- and IgG mAb-staining of hyphal secretion before and after periodate oxidation. Fungal secretions were treated 30 min with 0.1 M sodium periodate, dyalised by gel filtration and then compared to untreated secretion by SDS-PAGE and Western blot, followed by specific mannoprotein staining with digoxigenin-labelled Concanavalin A (Con-A) or the IgG anti-β1,3-glucan mAb. Lanes 1 and 3: untreated fungal secretion; lanes 2 and 4: periodate-oxidised secretion. Samples loaded onto the gel correspond to 25 µg polysaccharide. (0.71 MB TIF) [file pone.0005392.s001.tif]

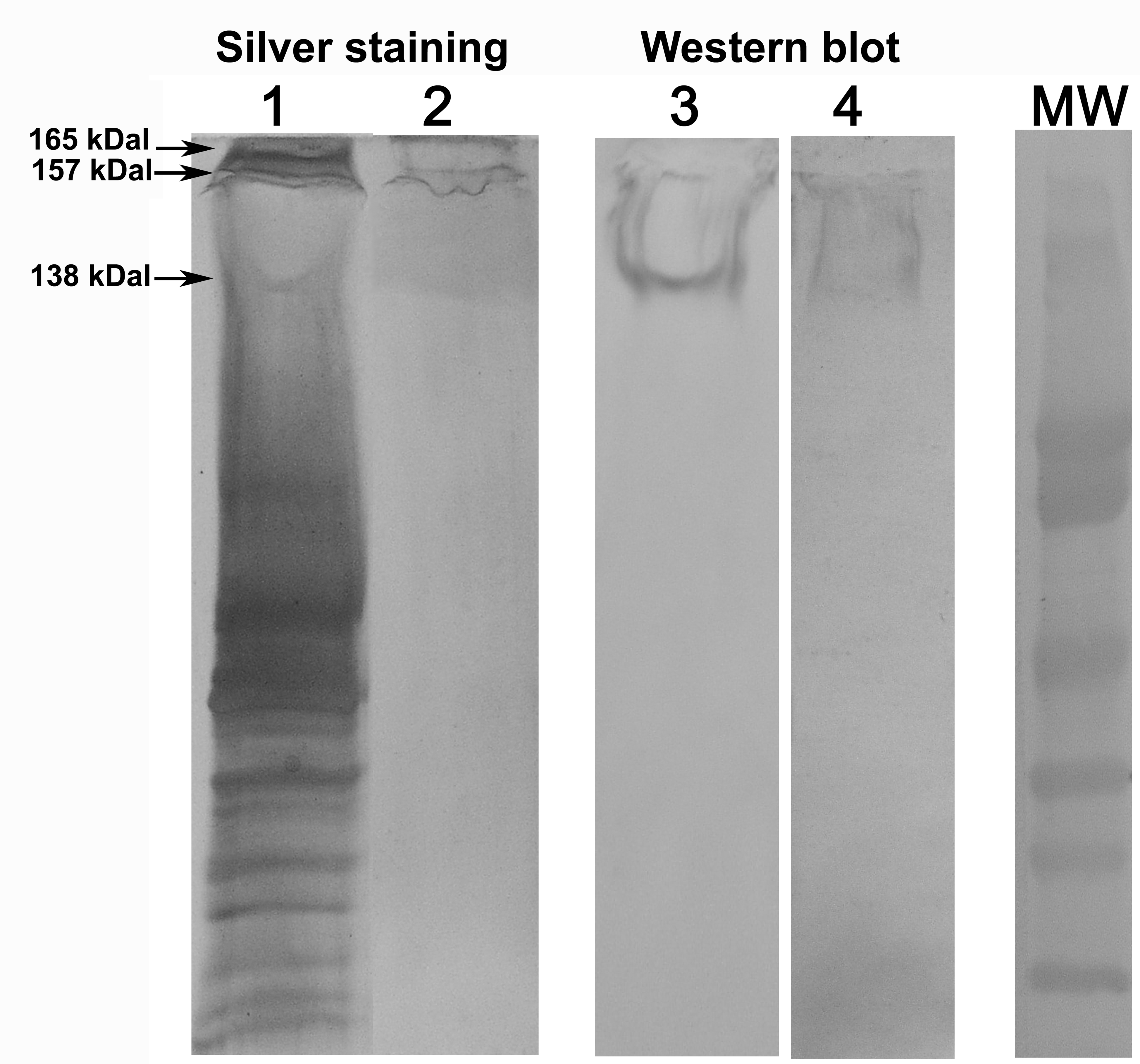

Supplement: Figure S2 — SDS-PAGE and Western blot analysis of IgG mAb-reactive material purified by immunoaffinity from hyphal secretion. Lane 1 and 2 show silver staining of the total hyphal secretion and the IgG mAb-immunopurified fraction, respectively. Lane 3 and 4 shows Western blot reactivity of the immunopurified fraction with the IgG mAb (Lane 3) or with the IgG fraction of a protective serum from mice immunized with the Lam-CRM vaccine (see text). (3.72 MB TIF) [file pone.0005392.s002.tif]
